# Mevalonate kinase deficiency: an updated clinical overview and revision of the SHARE recommendations

**DOI:** 10.3389/fimmu.2024.1466844

**Published:** 2024-11-12

**Authors:** Lilla Lengvári, Kata Takács, Anna Lengyel, Annamária Pálinkás, Carine Helena Wouters, Isabelle Koné-Paut, Jasmin Kuemmerle-Deschner, Jerold Jeyaratnam, Jordi Anton, Helen Jane Lachmann, Marco Gattorno, Michael Hofer, Nataša Toplak, Peter Weiser, Tilmann Kallinich, Seza Ozen, Véronique Hentgen, Yosef Uziel, Zsuzsanna Horváth, Márton Szabados, Paul Brogan, Tamás Constantin, Joost Frenkel

**Affiliations:** ^1^ Paediatric Centre, Semmelweis University, Budapest, Hungary; ^2^ Department of Pediatrics, University Hospitals Leuven, Leuven, Belgium; ^3^ Department of Pediatric Rheumatology, National Reference Centre for Rare Autoinflammatory Diseases and Inflammatory Amyloidosis, Centre Hospitalier Universitaire de Bicêtre, Assistance Publique-Hôpitaux de Paris, University of Paris Saclay, Le Kremlin-Bicêtre, France; ^4^ Pediatric Rheumatology, Department of Pediatrics and Autoinflammation Reference Center, University Hospital Tuebingen, Tuebingen, Germany; ^5^ Division of Woman and Infant, Wilhelmina Children’s Hospital, University Medical Center Utrecht, Utrecht, Netherlands; ^6^ Department of Pediatric Rheumatology, Hospital Sant Joan de De´ u, University of Barcelona, Barcelona, Spain; ^7^ National Amyloidosis Centre, University College London, and Royal Free Hospital London NHS Foundation Trust, London, United Kingdom; ^8^ UOC Reumatologia e Malattie Autoinfiammatorie, IRCCS Istituto G. Gaslini, Genoa, Italy; ^9^ Pediatric Rheumatology and Immunology, Hoˆ pital Riviera-Chablais, Rennaz, Switzerland; ^10^ Department of Allergology, Rheumatology and Clinical Immunology, University Children’s Hospital Ljubljana, University Medical Centre Ljubljana, Ljubljana, Slovenia; ^11^ Division of Rheumatology, Department of Pediatrics, University of Alabama at Birmingham, Birmingham, AL, United States; ^12^ Department of Pediatric Respiratory Medicine, Immunology and Critical Care Medicine, Charite´ – Universitätsmedizin Berlin, Berlin, Germany; ^13^ Department of Pediatric Rheumatology, Hacettepe University, Ankara, Türkiye; ^14^ French Reference Center for AutoInflammatory Diseases and Amyloidosis, Department of Pediatrics, Versailles Hospital, Versailles, France; ^15^ Pediatric Rheumatology Unit, Meir Medical Center, Tel Aviv University School of Medicine, Tel Aviv, Israel; ^16^ Inflammation & Rheumatology Section, UCL Great Ormond St Institute of Child Health, University College London, London, United Kingdom; ^17^ Division of Pediatrics, Wilhelmina Children’s Hospital University Medical Center Utrecht, Utrecht, Netherlands

**Keywords:** mevalonate kinase deficiency, diagnosis, genetics, treatment, guideline

## Abstract

Mevalonate kinase deficiency (MKD), a rare auto-inflammatory disorder, arises from mutations in the *MVK* gene, disrupting isoprenoid biosynthesis, and affecting cellular processes. This comprehensive review provides an updated perspective on MKD, including its aetiology, pathogenesis, diagnostic modalities, and therapeutic strategies. Based on recent research and clinical advances, our objective is to bridge the knowledge gaps in the 2015 SHARE guidelines. By describing molecular mechanisms, diagnostic dilemmas, and emerging therapies, this article should serve as a resource for clinicians and researchers, promoting a deeper understanding of MKD and guiding optimal patient care.

## Introduction

Mevalonate kinase deficiency (MKD) is a rare auto-inflammatory disorder (AID) characterized by a spectrum of clinical presentations that often intersect with other conditions and present diagnostic challenges. A timely and precise diagnosis of MKD is essential not only to initiate appropriate therapeutic interventions, but also to avoid the potential risks associated with misdiagnosis. The repertoire of therapeutic strategies for MKD is evolving rapidly. An updated and comprehensive review of these modalities is imperative to provide clinicians with a refined framework to improve patient outcomes and overall quality of life for those affected.

The true prevalence of MKD is unknown, while registries reported around 300 cases worldwide by 2013, the actual numbers are likely under-represented due to possible misdiagnosis or under-reporting, given the overlap of MKD symptoms with other inflammatory disorders (1). The prevalence was reported to be 0.39 per 1,000,000 person years in Germany in children ≤16 years by 2012, 1.3 per 1,000,000 for MKD in Eastern and Central European countries in children <19 years by 2010 and 5 per 1,000,000 of the general population in the Netherlands to 6.2 per 1,000,000 in Germany in children ≤16 years (2–4).

Although MKD has been recognized in all populations around the world, it appears to occur more frequently in individuals of Northern European heritage and only a few cases of MKD have been reported in Asian countries (5). The observed variance in geographic incidence suggests a genetic founder effect. Unique variants were identified in a Japanese cohort, which differed from those found in European patients, however, overall clinical presentations were similar in both groups (6). Additional cases have been reported from various ethnicities and regions around the world, indicating that the condition is not restricted to a specific geographic area. The broader distribution also emphasizes the importance of awareness and diagnostic capabilities across different regions to ensure the timely identification and management of MKD.

MKD arises from pathogenic variants of the mevalonate kinase gene (*MVK*) (7, 8). Although rare, this spectrum of diseases presents several clinical manifestations, from systemic disorders to localized skin pathologies. The pathophysiology of MKD is complex and rooted in genetic variations that disrupt critical biochemical pathways. Therefore, it is a metabolic autoinflammatory disorder (1). The *MVK* gene is central to the biosynthesis of isoprenoids, a class of lipids that play a variety of roles in cellular functions. Pathogenic variants in this gene lead to a deficiency of mevalonate kinase, which is the enzyme responsible for converting mevalonic acid to 5-phosphomevalonate. This disruption is the primary biochemical event that triggers the cascade of symptoms observed in MKD.

*MVK* is located on the long arm of chromosome 12 and encompasses 11 (10 coding) exons. Within these exons, various types of loss-of-function recessive variants are associated with MKD, e.g. missense single nucleotide variants leading to amino acid change, frameshifts leading to premature termination, intergenic deletions, etc. Specific variations are of such magnitude that they inhibit the formation of any functionally active enzyme. Interestingly, these null variants are not found in a homozygous form but are always paired in compound heterozygosity with less severe alterations. This pattern implies that a minimal degree of enzyme activity, however slight, is crucial for survival *in utero* (9).

More than 300 distinct *MVK* variants have been documented, and p.(V377I) and p.(I268T) mutations are notably prevalent among European populations (10). It is estimated that 1 in 65 people in the Dutch population carry a heterozygous pathogenic variant in the *MVK* gene. Among Dutch patients diagnosed with this disease, the frequency of the p.(V377I) allele is 42% (11).

Variants in the *MVK* gene can subtly alter the structure and stability of the mevalonate kinase enzyme, thus affecting its activity. The severity of the disease is correlated with a decrease in enzyme activity, often due to improper protein folding. Variants that affect highly conserved residues, especially within identified “hotspot” regions of the protein sequence (around residues 8–35 and 234–338), are more likely to cause significant structural changes, leading to more severe phenotypes. This insight suggests that pharmacological interventions aimed at stabilizing the *MVK* protein could offer potential therapeutic avenues for the treatment of MKD, especially for patients with severe forms such as mevalonic aciduria (MA) (12). Furthermore, the mevalonate kinase enzyme itself is thermolabile, and its activity decreases with increasing temperature. This attribute makes any febrile illness a potential trigger for autoinflammatory disease flares (13, 14).

The genotype-phenotype relationship in MKD is complex. Although the disorder is rooted in *MVK* variations, clinical manifestations can vary widely (15). This variability is not solely attributed to the type of variant; even individuals with identical genotypes can exhibit different clinical symptoms. The spectrum of MKD ranges from mild to severe autoinflammatory disease. The mild form is typically associated with variants that retain some residual enzyme activity (varies from 1.8% to 28%), leading to periodic episodes of fever and systemic inflammation. On the contrary, the severe form is often associated with alterations that result in little or undetectable enzyme activity (less than 0.5%), manifesting more pronounced neurological symptoms and developmental challenges (16).

The results of the Eurofever cohort confirm significant differences in the distribution of neurosensory defects between genotypic groups (17, 18). Severe gastrointestinal and musculoskeletal symptoms were found to be more prevalent in patients with genotypes excluding p.(Val377Ile) (10). On the other hand, the presence of the mild p.(Val377Ile) allele in a compound heterozygous or homozygous has been observed in ‘severe’ MKD, although anecdotally (19–21). Furthermore, a strong association was observed between severe neurological and ocular manifestations, particularly cataracts, and genotypes, including variants p.(Ala334Thr) and p.(Leu264Phe). An association between amyloidosis and compound heterozygosity for p.(Ile268Thr) and p.(Val377Ile) has also been also described (22).

Genetic testing, although instrumental, presents interpretative challenges. The Eurofever cohort, for example, may not comprehensively represent the larger MKD population. Acknowledging that such cohorts might encompass only a specific subset of individuals carrying particular genetic variants is imperative. Therefore, extrapolating phenotypic descriptions based on these selected cohorts to the broader population undergoing genetic testing could be erroneous. Taking into account these intricacies, a judicious approach to the interpretation of genetic data is advocated, ensuring that conclusions are drawn in accordance with the broader clinical and genetic context of MKD (10).

In rare cases where only one variant is detected, it is essential to consider the possibility of an undetected second variation; or the individual merely being a heterozygous carrier and the observed genotype not being the cause of the phenotype being investigated. Some individuals with a single detected variant may still have symptoms. However, in the context of MKD, this is rare (10).

A distinct disorder caused by *MVK* variants is diffuse superficial actinic porokeratosis (DSAP). In this rare skin disorder, dominant germline or somatic *MVK* variants have been identified (23, 24). Second-hit somatic variants were recently described in DSAP lesions and are a likely explanation for the acquisition of these skin lesions in areas exposed to the sun (20, 23). As DSAP is clinically distinct from systemic MKD, it will not be further discussed in this article.

Environmental factors, such as infections, vaccinations or stress, can also influence the onset and severity of symptoms of MKD. Additionally, other genetic factors or modifier genes can play a role in determining the phenotype, adding another layer of complexity to the genotype-phenotype correlation (25, 26). Interestingly, the *MVK* gene shares a conserved promoter region with the *MMAB* gene, suggesting potential epistatic interactions. Recent studies have highlighted the importance of considering both the *MVK* and *MMAB* genes in the molecular diagnosis of MKD. A specific genetic variation (rs1450500) in *GRID2* in the 4q22.2 chromosome region was found to associate with MKD, possibly as a phenotype modifier (15, 27).

In patients with very early-onset severe inflammatory bowel disease (IBD), particularly those with a history of recurrent or chronic fever, peritoneal adhesions, and atypical pathology of IBD, MKD should be a differential consideration (28). A recent study highlighted a shared genetic background between early-onset IBD and MKD. Children who develop intestinal inflammation at an early age may have different phenotypes and genetic architectures. Although some of these children align with the classical categorization of IBD, others fit the profile of monogenic diseases such as MKD. This overlapping genetic foundation could explain the shared clinical and phenotypic characteristics observed in both conditions (29).

### Clinical spectrum of mevalonate pathway disorders

Genetic mutations that disrupt the mevalonate pathway can result in a variety of metabolic and inflammatory disorders (**Table 1**). MKD is the primary example, manifesting a spectrum of clinical presentations ranging from mild forms, formerly known as hyper IgD syndrome (HIDS), to severe forms such as mevalonic aciduria (MA). (We will use the taxonomy of the consensus proposal for autoinflammatory diseases, which suggests using ‘mild’ MKD for HIDS and ‘severe’ MKD for MA (30)). In this paper, we will focus on MKD, but it is important to note that conditions such as porokeratosis, phosphomevalonate kinase deficiency (PMKD), and macrophage activation syndrome (MAS) are also related to abnormalities in the mevalonate pathway.

**Table 1:** Clinical spectrum of mevalonate pathway disorders.

Syndrome	Genetic Basis	Clinical Manifestations	Prognosis	References
Mevalonic aciduria (MA)	Mutations in the *MVK* gene leading to deficiency in cholesterol and isoprenoid biosynthesis	Developmental delays, hypotonia, cerebellar ataxia, peripheral neuropathy, systemic inflammation, neurological complications	Potentially fatal, early diagnosis and comprehensive management essential	(4, 97)
Hyperimmunoglobulinemia D syndrome (HIDS)	Mutations in the *MVK* gene leading to recurrent but less intense inflammatory episodes	Recurrent fever, lymphadenopathy, gastrointestinal disturbances, skin rash, joint involvement	Generally favourable, normal life expectancy with medical treatment and support	(4, 98)
Phosphomevalonate kinase deficiency (PMKD)	Mutations in *PMVK* gene, systemic symptoms similar to HIDS	Recurrent fevers, arthritis, rash, abdominal pain, diarrhoea, increased IL-1β secretion	Emerging condition, need for further evaluation in patients with HIDS symptoms	(99–101)
Porokeratosis	Associated with MKD, benign but risk of malignant transformation	Small, slowly enlarging plaques with a raised border, various skin manifestations	Regular dermatological evaluations necessary, monitor for changes	(20, 24, 102)
Macrophage activation syndrome (MAS)	Excessive activation and proliferation of specific immune cells	Widespread inflammation, multiorgan dysfunction	Life-threatening, awareness and prompt recognition crucial	(103–107)

### Update on the 2015 SHARE guidelines

The 2015 SHARE initiative provided a detailed overview of AIDs, synthesizing the data available up to 2013 (31). (For the purposes of citation and clarity, this resource is henceforth referred to as the “2015 SHARE guideline”). A decade later, the MKD research landscape has seen significant advances that call for an updated and evidence-based perspective on MKD. In particular, advancements in genetic databases like Infevers have enriched the understanding of pathogenic variants, improving diagnostic accuracy. The increasing accessibility and precision of next-generation sequencing (NGS) has enabled earlier and more reliable diagnosis, while novel diagnostic tools, such as mevalonate kinase enzyme activity assays, are now available. These tools are especially impactful in cases with severe phenotypes, where they can influence critical clinical decisions, such as the need for bone marrow transplantation.

The updated recommendations address these diagnostic challenges by emphasizing the integration of genetic and biochemical testing for greater diagnostic confidence. In terms of therapy, recent studies on the long-term outcomes of biologic therapies, especially IL-1 inhibitors, have informed this revised guideline. Additionally, the updated recommendations incorporate new evidence on the safety and efficacy of vaccinations for MKD patients receiving treatment, thereby offering a more comprehensive approach to patient care.

## Methods

Our objective was to review the literature to update the findings of the 2015 SHARE expert group review of MKD (31).

### Search strategy

A comprehensive search was conducted on PubMed to identify relevant articles published after the last SHARE review. To maintain consistency in the type of articles retrieved, the search terms were derived from the original SHARE review. Additionally, index terms, PICOs, and other relevant search parameters were applied to ensure an inclusive approach, capturing all potentially relevant studies related to MKD. The detailed search strategy, including the specific terms and PICOs used, is provided in the **Supplementary Materials**.

### Study selection

The titles and abstracts of recovered articles were initially selected by AP and TC to identify potentially relevant studies. TC and ZSH conducted subsequent full-text evaluations to determine eligibility. Any uncertainties or disagreements about the inclusion of the study were resolved by discussion and consensus. The initial search yielded 407 publications. Title and abstract screening excluded 279 papers. Based on full-text analysis, an additional 68 publications were excluded. Papers were excluded if they were case reports with fewer than three patients, were not original articles, or contained irrelevant information.

### Data extraction

Once the studies were selected for inclusion, AN and LL performed data extraction using a standardized data extraction form. This form captured the essential details of the study design, population, interventions, outcomes, and critical findings. The collaborative approach ensured the consistency and comprehensiveness of the information collected.

### Quality assessment

To evaluate the risk of bias in the included studies, we conducted a thorough risk analysis using the ROBINS-I (Risk Of Bias In Non-randomized Studies - of Interventions) tool. This tool allowed for a structured assessment of bias across seven domains: confounding, selection of participants, classification of interventions, deviations from intended interventions, missing data, measurement of outcomes, and selection of reported results. Each domain was carefully examined to ensure a comprehensive evaluation. Two independent reviewers, LL and KT, assessed the risk of bias. The reviewers discussed any disagreements until a consensus was reached. The detailed results of the risk of bias assessment are provided in the **Supplementary Materials**.

### Validity assessment

We adopted the Levels of Evidence and grading recommendations described by Burns et al. (32). Each study was classified according to a hierarchical system of levels of evidence, ranging from Level I (high-quality randomized controlled trials) to Level IV (expert opinion and case reports). The strength of recommendations was graded based on the quality of evidence, with strong recommendations (Grade A) supported by Level I evidence or consistent findings from multiple studies of levels II, III, or IV, while weaker recommendations (Grade C) were based on inconsistent or lower-level evidence (KT, LL, MSZ). The results of this assessment are provided in the **Supplementary Materials**.

### Data synthesis

A select working group (TC, PB, SO, MG, JF) developed a set of proposed recommendations based on the available evidence. Subsequently, these recommendations were submitted to the authors of the original SHARE manuscript for their evaluation and online voting on the level of agreement. A consensus was deemed to be reached if the level of agreement exceeded 7/9 points, obviating the need for further consultation. The table of recommendations includes the degree of supporting evidence and the level of agreement for each recommendation.

## Recommendations

Management of MKD requires a comprehensive and multidisciplinary approach to ensure optimal patient outcomes (**Table 2**). To facilitate this, several overarching statements have been developed to guide clinicians in the diagnosis and management of MKD. These statements emphasize the importance of managing patients within a multidisciplinary team led by experts in hereditary autoinflammatory disorders. Such teams should ideally include rheumatologists, immunologists, geneticists, and nurse specialists to provide a holistic approach to care. Integrating genetic counselling within this framework is also crucial. Furthermore, educating patients and caregivers about targeted therapies, lifestyle changes, and the importance of regular follow-up is essential for effective disease management. Empowering patients through resources and support networks is recommended to improve self-management capabilities. Lastly, encouraging patient participation in clinical studies or registries is vital to advance understanding and treatment of MKD.

**Table 2:** Overarching Statements.

Overarching statements:	LoE	LoA (1–9) mean ± SD
1. Patients diagnosed with MKD should be managed within a multidisciplinary team framework led by clinicians with expertise in hereditary autoinflammatory disorders.	**A**	**8,2 ± 2,0**
a. This specialised care team should ideally include, but not be limited to, rheumatologists, immunologists, geneticists, and nurse specialists who can offer a multidisciplinary approach to diagnosis, treatment, and long-term management.	**A**	**7,4 ± 2,2**
b. Integrating genetic counselling into this care model is imperative.	**A**	**7,9 ± 1,8**
2. Provide comprehensive education to patients and caregivers:	**A**	**-**
a. Patients and caregivers should be well informed about the role of targeted therapies, the importance of lifestyle adjustments to manage disease triggers, and the necessity of regular medical follow-up to monitor disease progression and response to treatment.	**A**	**8,1 ± 1,6**
b. Providing resources and support networks can also empower patients in self-management of their condition.	**A**	**8,3 ± 1,1**
3. Encourage patients to participate in clinical studies or registries to advance understanding of MKD and future treatments.	**B**	**8,7 ± 0,5**

Level of evidence (LoE): A: Level I evidence or consistent findings from multiple studies of levels II, III, or IV; B: Levels II, III, or IV evidence and findings are generally consistent; C: Levels II, III, or IV evidence, but findings are inconsistent; D: Level V evidence: little or no systematic empirical evidence; Level of agreement (LoA).

## Diagnosis

### Laboratory investigations

Currently, there is no single laboratory test that provides a definitive diagnosis (**Table 3**). Markers of inflammation are commonly assessed in clinical settings, especially during flare-ups. Serum levels of C-reactive protein (CRP) and serum amyloid A (SAA) increase during inflammatory flare-ups in MKD. However, SAA may offer a more sensitive measure of inflammation in MKD. In some cases, SAA remains elevated even when CRP levels normalize, suggesting ongoing inflammation that may not be captured by CRP alone. SAA levels can be used to monitor the efficacy of MKD treatments. Although amyloidosis is a rare complication in MKD, patients with chronically elevated SAA levels may be at risk for amyloidosis (4, 33); therefore, regular monitoring of the SAA level can help identify at-risk patients and initiate early interventions.

**Table 3:** Recommendations for the diagnosis of MKD.

Diagnosis:	LoE	LoA (1–9) mean ± SD	
Laboratory investigations:			
1. Prioritize MVK gene testing for its crucial role in establishing a definitive diagnosis of MKD, as it significantly contributes to confirming the condition.	**B**	**8,2 ± 1,6**
2. However, other laboratory findings should also be considered together with clinical observations, especially in patients with negative results of genetic tests due to potentially yet unrecognised mutations.	**B**	**7,7 ± 2,2**
3. Verify inflammation through laboratory tests, for example total WCC, ESR, CRP, SAA during attacks. These are not diagnostic of MKD but will confirm systemic inflammation; note that these usually vary (and may normalize) between flares.	**A**	**8,13 ± 1,4**
4. IgD-level testing is not recommended due to its negligible diagnostic value.	**B**	**8,67 ± 0,6**
5. Urinary mevalonic acid levels are usually the highest during flares. Nevertheless, normal urinary mevalonic acid level alone has a low negative predictive value and does not negate the possibility of MKD and, therefore, is not mandatory before advancing to genetic testing.	**B**	**7,47 ± 2,1**
6. It is recommended that enzyme activity assays be reserved for cases involving patients with particularly severe phenotypes, where the results may significantly influence the clinical decision, particularly in evaluating the need for bone marrow transplantation. This assay is conducted only in a limited number of specialised laboratories, thus limiting its availability as a routine diagnostic test.	**B**	**7,53 ± 1,5**
Clinical classification and guiding genetic investigations (clinical parameters as predictors):	LoE	LoA (1-9) mean ± SD	
1. Clinical diagnostic scores and classification criteria can be used as supportive tools to evaluate patients with suspected hereditary autoinflammatory syndromes, such as MKD.	**A**	**7,9 ± 1,1**	
2. However, genetic tests are now much more widely available and should be performed if the clinical diagnosis is suspected, regardless of a clinical predictive score/classification result.	**B**	**8,4 ± 1,3**	
2a. Consider genetic testing regardless of scores: even if diagnostic scores are negative, consider genetic testing when clinical suspicion persists.	**A**	**7,4 ± 2,1**	
2b. Maintain a holistic approach to patient evaluation. Consider all clinical presentations and history fully, as these syndromes often present with a range of symptoms that may not be fully captured by diagnostic scores.	**A**	**8,4 ± 1,3**	
Genetic testing	LoE	LoA (1-9) mean ± SD	
1. Conduct genetic testing for all suspected cases of MKD to confirm/refute the diagnosis.	**B**	**7,9 ± 1,4**	
2. Consult “The Registry of Hereditary Auto-inflammatory Disorder Mutations” database to assess the pathogenicity of identified genetic variants (https://infevers.umai-montpellier.fr/web/).	**A**	**8,3 ± 0,7**	
3. Ensure that genetic results are interpreted in the context of patient inflammatory symptoms, engaging an auto-inflammatory expert for an accurate assessment.	**B**	**8,6 ± 0,5**	
4. Provide genetic counselling to patients and their families.	**A**	**8,5 ± 1,1**	
5. For patients with clinical signs of MKD but with negative or inconclusive genetic testing, consider additional genetic tests, including searches for deletions or duplications in the MVK gene.	**B**	**7,8 ± 1,9**	
6. Patients with negative results of genetic tests should be classified as having an undifferentiated or unclassified autoinflammatory disorder (uAID), and their treatment should follow the best practices established for uAID.	**A**	**7,6 ± 1,9**	
7. For patients with negative genetic results, consider consulting other specialist centres to ensure the most informed approach to further investigation and treatment.	**A**	**7,7 ± 1,7**	

Level of evidence (LoE): A: Level I evidence or consistent findings from multiple studies of levels II, III, or IV; B: Levels II, III, or IV evidence and findings are generally consistent; C: Levels II, III, or IV evidence, but findings are inconsistent; D: Level V evidence: little or no systematic empirical evidence; Level of agreement (LoA).

While serum immunoglobulin evaluation, focusing primarily on IgD and IgA, can aid in suspecting MKD, caution is advised. Elevated IgD levels can support a provisional diagnosis when aligned with clinical manifestations, but they are frequently elevated in other autoimmune diseases such as SLE (34). Additionally, relying solely on clinical symptoms and IgD levels for diagnosing MKD has notable pitfalls (17). IgD levels do not consistently correlate with disease activity, enzyme functionality, or genotype in MKD (35–37). Given that some patients exhibit normal IgD levels, the diagnostic sensitivity is limited (36, 38). More than two-thirds of patients show elevated IgA levels (39).

The detection of mevalonic acid in urine samples may be of great importance for diagnosis. Urinary mevalonic acid levels are highly informative during symptomatic periods of MKD, with high sensitivity and specificity (16). Although patients with severe phenotypes consistently show high levels, those with the milder phenotype typically exhibit elevated levels only during flare-ups. Therefore, the urine sample must be taken during fever if this laboratory test is available. This test, especially when using advanced techniques such as gas chromatography and mass spectrometry, can provide a more specific indication of MKD (40). While normal mevalonic acid excretion significantly reduces the likelihood of MKD, indicated by a 98% negative predictive value, it does not completely eliminate the possibility. Consequently, it is an effective screening tool when genetic testing is unavailable. However, elevated urinary mevalonic acid levels warrant further confirmatory testing through *MVK* gene analysis, given their 71% positive predictive value, which requires additional diagnostic steps. In summary, even when urinary mevalonic acid excretion is positive, analysis of the *MVK* gene remains essential to confirm the diagnosis of MKD diagnosis (16).

Although less routinely assessed than the above markers, emerging evidence suggests that impaired protein prenylation has potential diagnostic value. Assessing prenylation defects in PBMC lysates can offer additional information, especially when genetic tests are inconclusive (41–43).

Enzyme activity measurements are the gold standard for diagnosing MKD. In cases where the genotype is known to be pathogenic, genetic testing is equally reliable. However, when one or both alleles of *MVK* have variants of unknown significance, functional tests are essential to determine their pathogenicity. Urinary mevalonic acid levels can be informative in such cases, although enzyme activity measurements are superior. However, the latter requires shipping samples to specialized laboratories.

### Clinical classification and guiding genetic investigations (clinical parameters as predictors)

Determining which patients deserve genetic testing based on clinical presentation remains vital in hereditary autoinflammatory syndromes. Gattorno et al. identified clinical parameters that predict gene mutations associated with these syndromes. The derived diagnostic score, based on indicators such as age of onset, familial history of periodic fever, thoracic pain, abdominal discomfort, diarrhoea, and oral aphthosis, showed strong sensitivity and specificity to guide genetic testing decisions (44). However, the authors of the SHARE recommendation felt it essential to emphasize that this score can help select children for further diagnostic tests, but that a negative score should not exclude (genetic) testing (31, 45).

In 2015, as part of the Eurofever project (utilizing patient data from the Eurofever database), the first set of international classification criteria for MKD was published. These criteria marked a significant milestone in the field, providing a standardized framework for identifying and classifying this condition. This scoring system is based on the clinical manifestations observed during typical inflammatory episodes, excluding the influence of intercurrent infections or other comorbidities. For MKD, the criteria included: onset age less than 2 years (awarded 10 points), presence of aphthous stomatitis (11 points), generalised lymph node enlargement or splenomegaly (8 points), painful lymph nodes (13 points), occasional or frequent diarrhoea (20 points), persistent diarrhoea (37 points), and absence of chest pain (11 points). A cumulative score of 42 points or higher yielded a sensitivity of 93% and a specificity of 89% in the validation cohort. Understanding that these criteria are intended to guide further diagnostic tests in patients suspected of having an autoinflammatory disease is crucial. However, they should only be used after ruling out other possible causes of fever and inflammation, such as infections, immunodeficiencies, malignancies, or other immunological conditions (46). Patients with MKD typically show symptoms in the first year of life. The German and international (EULAR/ACR) guidelines emphasise this crucial time frame. In particular, the suspicious age of onset was reduced to 1 year, facilitating early diagnosis and treatment. This proactive measure ensures timely intervention for these young patients, optimising their chances of better outcomes (47, 48).

However, these criteria do not consider the results of genetic analyses, which are now an essential tool to accurately diagnose and classify hereditary recurrent fevers (HRF). In a small cohort, the Eurofever classification criteria (from 2015) were not correlated with the final clinical diagnosis of patients diagnosed with MKD; however, they were associated with CAPS and TRAPS (49).

Gattorno et al. conducted a comprehensive study to develop and validate new classification criteria for HRF and periodic fever, aphthosis, pharyngitis, and adenitis (PFAPA). Using a multistep approach, the team incorporated clinical, laboratory, and genetic variables from the Eurofever Registry. Specifically, a random sample of 360 patients was selected, with 60 patients representing each disease category: FMF, TRAPS, MKD, CAPS, PFAPA, and undefined recurrent fever (uRF). This methodology was further refined through consensus conferences and rigorous statistical analysis. The culmination of this effort produced criteria that exhibited high sensitivity and specificity for HRF and PFAPA. In the context of MKD, the diagnosis is anchored primarily in identifying biallelic mutations of *MVK* gene mutations. The expert panel proposed a classification system for patients with MKD based on the number of *MVK* mutations identified. Patients with two identified *MVK* mutations were classified as ‘biallelic MKD’, while those with a single mutation were termed “mono-allelic MKD” (50).

The criteria were validated on real-life data by two working groups. The first study, using data from 455 patients from France and Switzerland, confirmed the high precision of the new classification criteria, especially for patients with a confirmatory genotype. However, the clinical criteria designed for patients without genetic test results or those without genetic abnormalities showed limitations in performance. This validation underscores the importance of combining clinical and genetic data to accurately classify hereditary auto-inflammatory diseases. Note that the classification criteria had excellent specificity for CAPS and TRAPS (98% specificity each), fair specificity for FMF (88%), but poor specificity for MKD (58%) (51). A Turkey-based working group has revealed that the genetic variations prevalent in specific populations can affect the observable traits of some patients. This challenges the accurate classification of such individuals using current criteria. As a result, in populations with common genetic variations, the classification criteria should be adjusted to better describe the clinical characteristics (52).

It is important to note that while these classification criteria are necessary for patients to be included in research studies, they are not meant to be used as standalone diagnostic criteria.

### Genetic tests

Molecular genetic tests are recommended for all patients. The importance and feasibility of genetic testing in diagnosis has increased because of advancements in genetic analysis systems. Distinct disparities can exist between the AID phenotype and genotype, with not all genetic alterations being pathogenic (10). The ‘infevers’ database serves as a resource to determine the current pathogenicity of the genetic variant (53). The identification of the pathogenic variant supports the clinical diagnosis (47).

Experts from the SHARE initiative have indicated that interpreting the results of genetic tests can present difficulties. In the field of hereditary recurrent fevers (HRF), it is crucial to recognise that classic clinical symptoms do not always align with typical genetic mutations. A recent cohort underscored this discrepancy: Of 15 genetically confirmed cases, only one exhibited both a classical clinical presentation and the expected *MVK* gene mutation indicative of MKD (54). Furthermore, HRF can manifest even when genetic tests yield negative results. This highlights the complexity of HRF diagnosis and the potential challenges in correlating clinical presentations with genetic findings.

Therefore, the interpretation of certain genetic variants should be made in the context of the inflammatory phenotype, preferably by an expert in the field (48). The guidelines for the genetic diagnosis of AID offer direction for both doctors and geneticists (55). The SHARE group advocated for the application of such guidelines during the diagnostic procedure (31).

According to the best-practice guidelines of ISSAID/EMBQN, when there is a clear clinical suspicion of MKD, it is advisable to initiate the diagnostic process by sequencing all exons of the *MVK* gene using Sanger or NGS techniques. Should these sequencing efforts yield no definitive results, the next step involves the advancement of CNV detection. Array comparative genomic hybridization (array-CGH) is recommended to identify large-scale genomic alterations, while for small variants, alternative genetic testing approaches such as multiplex ligation-dependent probe amplification (MLPA) are recommended, although these specialised tests are typically available only in specialised centres (56).

The SHARE article explored next-generation sequencing (NGS) as a potential tool for diagnosing atypical autoinflammatory syndrome in children. Although there was not enough evidence to recommend NGS, the authors suggest that it may become valuable in the future. Furthermore, the article emphasised the importance of establishing clear diagnostic criteria and definitions for atypical autoinflammatory syndrome. Diagnosing autoinflammatory diseases can be challenging because the clinical findings of some patients may indicate a specific disease, but overlapping symptoms make it difficult to confirm. Furthermore, a minimum of 40% of people who are likely to experience an autoinflammatory condition do not align with any recognised disease (57). In these cases, it is recommended to use panel tests consisting of at least eight genes (56).

In a scenario where strong clinical suspicion of autoinflammatory disease (AID) prevails, but symptoms do not clearly correlate with any of the known autoinflammatory diseases, it is recommended to perform an NGS panel test. This step is particularly crucial to broaden the diagnostic lens and potentially identify new or less common genetic variants associated with autoinflammatory conditions (56).

## Patients with symptoms suggestive of MKD but negative genetic testing

Patients with symptoms of MKD but negative genetic testing should only be classified as having undifferentiated auto-inflammatory disease (uAID) (58) after alternative monogenetic or complex (i.e. non-genetic diseases such as Behcet’s) autoinflammatory diseases have been excluded. The term uAID can be useful in clinical practice, as it allows clinicians to define cases with vague and overlapping features that may have an autoinflammatory aetiology. This diagnosis implies that the patient’s condition must be regularly reviewed for any evolution into a more defined clinical entity. With advances in genetic testing and ongoing research, there is hope that more precise diagnoses and treatments will be found for these patients (59).

There is evidence that targeted therapy may still be effective for patients with uAID. For example, a study evaluating the safety and efficacy of canakinumab, an anti-IL-1β agent, in patients with uAID showed that the patients had positive responses to treatment (60). In addition, anakinra, an IL-1 receptor antagonist, has been shown to be effective in patients with uAID. It was used both as a diagnostic challenge and as a treatment, indicating that IL-1 dysregulation may play a role in the pathogenesis of the disease of these patients (61, 62).

Integrating the patient into a registry of complex or undiagnosed autoinflammatory conditions is also recommended, which can provide information for future research and possible treatment pathways (63).

## Treatment

### Anti-inflammatory treatment

The treatment of MKD is multifaceted, as highlighted by several guidelines, including SHARE, EULAR/ACR, and the German guidelines (31, 47, 48) (**Table 4**). The primary objective of treatment is to alleviate acute symptoms during inflammatory episodes and to reduce the frequency and severity of these episodes.

**Table 4:** Recommendations for the management and treatment strategies of MKD.

Treatment:	LoE	LoA (1-9) mean ± SD
Anti-inflammatory treatment		
1. The mainstay of treatments is with inhibitors of the interleukin-1 (IL-1) pathway, such as canakinumab (registered) or anakinra (off label), to treat and prevent inflammatory episodes.	**A**	**8,3 ± 1,5**
2. Compared with other autoinflammatory diseases, higher doses of canakinumab may be required to control MKD flares.	**A**	**8,5 ± 0,7**
3. On-demand IL-1 blockade with anakinra may be considered on a case-by-case basis.	**B**	**8,2 ± 0,9**
4. NSAIDs may be used to for symptomatic relief of mild disease flares.	**B**	**8,2 ± 0,9**
5. Short-term glucorticoids can be used to treat disease flares.	**B**	**8,1 ± 0,9**
6. Consider TNF or IL-6 blockade as potential second-line biologic agents for patients who do not respond adequately to interleukin-1 inhibitors.	**B**	**7,9 ± 1,0**
7. Statins or bisphosphonates for MKD treatment are not recommended.	**C**	**8,7 ± 0,5**
8. Allogeneic hematopoietic stem cell transplantation for severe refractory cases of MKD remains a viable therapeutic option but should be balanced against the risks involved.	**A**	**8,5 ± 0,6**
Treat-to-target strategies:	LoE	LoA (1-9) mean ± SD
1. Treat to the following targets: reduced frequency and severity of disease flares; normalization of acute phase reactants; limitation of glucocorticoid toxicity. Growth and patient quality of life are usually optimized if these are achieved.	**A**	**8,3 ± 0,9**
2. Consider using evaluations and tools such as the autoinflammatory disease activity index (AIDAI), the physician’s global assessment and the patient’s parent global assessment to monitor clinical disease activity, ensuring timely treatment adjustments based on therapeutic efficacy.	**A**	**8,4 ± 0,9**
3. Regular serological tests (every 3 months as a minimum) of acute phase markers (WCC, CRP, and SAA) should be used to monitor serological disease activity to detect subclinical inflammation.	**A**	**7,9 ± 1,2**
4. Regular monitoring of urinalysis for microalbuminuria or protein to creatinine ratio is recommended.	**A**	**7,9 ± 1,4**
5. Monitor growth regularly (every 3-6 months).	**A**	**8,2 ± 1,1**
6. Consider the escalation of therapy if these targets are not being met.	**A**	**8,1 ± 1,1**
Vaccination:	LoE	LoA (1-9) mean ± SD
1. Patients with MKD should adhere to their vaccination schedule where possible, as the potential risk of a disease flare is outweighed by the serious consequences of vaccine-preventable infections.	**A**	**8,5 ± 0,7**
2. The vaccination strategy is dictated primarily by the immunomodulatory treatments used, rather than by the disease itself. It is recommended to consult international and national guidelines, specifically in relation to the receipt of live vaccines while on biologic treatment.	**A**	**7,9 ± 2,1**
3. Before receiving vaccines, patients with MKD should consult with their healthcare provider, ideally a specialist familiar with autoinflammatory diseases, to discuss the benefits and risks associated with vaccination in the context of their condition.	**A**	**8,2 ± 1,8**
4. In managing flares for MKD patients around vaccination times, NSAIDs rather than glucocorticoids can be specifically suggested for symptomatic relief to avoid significantly dampening the response to vaccines.	**D**	**7,9 ± 1,7**

Level of evidence (LoE): A: Level I evidence or consistent findings from multiple studies of levels II, III, or IV; B: Levels II, III, or IV evidence and findings are generally consistent; C: Levels II, III, or IV evidence, but findings are inconsistent; D: Level V evidence: little or no systematic empirical evidence; Level of agreement (LoA).

Non-steroidal anti-inflammatory drugs (NSAIDs) are often the first line of treatment for acute inflammatory episodes in patients with MKD. Both the SHARE and German guidelines recommend NSAIDs as the initial intervention for acute episodes, and EULAR/ACR echo this sentiment. However, in practice NSAIDs alone usually do not provide adequate relief for inflammatory attacks.

For those who exhibit limited response to NSAIDs or experience more pronounced symptoms, corticosteroids are an alternative. Administering short-term corticosteroids promptly after the onset of inflammatory symptoms can effectively mitigate these manifestations. This strategy is supported by the three guidelines, including the German guidelines that highlight the possible side effects of prolonged use of corticosteroids and the need for vigilant monitoring (31, 47, 48).

Biological agents, particularly those targeting interleukin-1, have become crucial in the treatment of MKD. Anakinra, an interleukin-1 receptor antagonist, is approved for CAPS, FMF, and DIRA; however, for MKD, it is still off-label. According to a recent review of 21 patients, the response rate to anakinra in paediatric patients with HIDS was 90%, while that of etanercept was 50% (64). In its retrospective analysis, the Eurofever Registry observed 19 patients receiving continuous anakinra treatment. Of these, 13 showed a partial response, three had a complete response, and treatment was ineffective for three others. Furthermore, eight patients used anakinra exclusively during flare-ups, resulting in complete relief for three and partial relief for five (22). The sustained prophylaxis and on-demand treatment is promoted by SHARE, EULAR/ACR, and the German guidelines (31, 47, 48). For on-demand use, it is crucial to initiate anakinra at the first signs of an episode, which can reduce its duration. However, without robust evidence, on-demand anakinra is best reserved for patients with sporadic episodes (65), with clear documentation of absence of acute phase response in between attacks. This study also showed that although treatment resulted in a decrease in CRP levels, the duration of the fever, and the severity of symptoms, it did not reduce the frequency of fever episodes. Therefore, for patients who experience episodes more frequently than once every 4-6 weeks, the transition to continuous treatment is advised (66). Anakinra is administered daily subcutaneous injections, sometimes more often; however, some patients may perceive this frequent regimen as challenging due to the pain associated with both the injection and the medication itself. The main adverse events associated with anakinra treatment are reactions at the injection site, particularly in children (67). These reactions are mostly considered mild medically; some can resolve over time, but they are potentially concerning for the family. Therefore, engaging in a comprehensive dialogue with the patient, considering their fears and preferences, is crucial to optimise adherence to treatment.

Canakinumab (CAN), a neutralizing monoclonal antibody against interleukin-1β, offers the advantage of less frequent dosing because of its longer half-life, which can improve patient compliance and overall quality of life. It has shown efficacy in HIDS by decreasing both the frequency and intensity of inflammatory episodes and has obtained EMA and FDA approval for MKD.

CAN treatment has shown promising results in several international observational registries/studies (66–70). Two clinical investigations were conducted to assess the effectiveness and safety of CAN in patients with active MKD. The initial study, an open-label Phase II trial, was led by Arostegui et al. Their findings indicated that canakinumab was both useful in the management of active MKD and successful in inhibiting inflammation-associated transcriptional reactions.

The CLUSTER study by Benedetti et al. represents a pivotal randomised controlled trial (RCT) in the field of MKD research. In this study, the efficacy of CAN was rigorously evaluated in patients with MKD. Participants with genetically confirmed MKD were randomised to receive 150 or 300 mg of canakinumab subcutaneously or a placebo every 4 weeks. The primary endpoint was a complete response by week 16, which was characterised by the resolution of the flare without recurrence. The results were promising for patients with MKD receiving canakinumab. At week 16, 35% of the MKD patients treated with CAN achieved a complete response, in stark contrast to the 6% response rate observed in the placebo group. This significant difference underscores the potential of canakinumab as an effective therapeutic intervention for MKD. In particular, although infections were the most frequently reported adverse events among CAN recipients, the study findings suggest a favourable risk-benefit profile for the drug in the context of MKD management.

The extended open-label phase 3 CLUSTER trial provided a comprehensive evaluation of canakinumab efficacy and safety over a 72-week period in patients with MKD. Administered at doses of 150 or 300 mg at 4- or 8-week intervals, CAN resulted in a significant reduction in disease flare-ups. 64% of the participants did not experience flares during the study duration, a substantial improvement from their baseline median of 12 annual flares. At the end of the study, more than 90% of the subjects exhibited minimal or absent disease activity and consistent reductions in inflammatory markers, such as CRP, were observed. The adverse events matched expectations, strengthening the potential of CAN as a viable long-term therapeutic option for the management of MKD (71).

In a subsequent analysis of the CLUSTER trial, Lachmann et al. investigated the health-related quality of life (HRQoL) of patients with MKD treated with CAN. The study underscored the profound burden of uncontrolled MKD, with patients who had previously experienced a median of 60 days a year with active disease symptoms. Using tools such as CHQ-PF50 for paediatric patients and SF-12 for adults, the study revealed marked improvements in HRQoL during CAN treatment. This improvement in well-being, both physically and psychosocially, further solidifies the potential of canakinumab as a crucial therapeutic option for patients with MKD/HIDS (72).

In a study by Balci et al., the impact of CAN treatment on growth parameters was examined in children with autoinflammatory diseases. Their research included 24 patients, of whom 9 were diagnosed with MKD. The research highlighted that after CAN treatment, there was a significant increase in the mean height, weight and BMI SD scores of the patients. This improvement in growth parameters was attributed to the suppression of ongoing inflammation due to the efficacy of the drug in controlling disease activity. In particular, growth parameters after treatment did not show significant differences based on sex, age at diagnosis, or duration of diagnostic delay. This study underscores the potential benefits of CAN in improving growth outcomes in children with autoinflammatory conditions, including MKD (73).

In an observational study by Hosono et al., the safety and efficacy of CAN for the treatment of MKD were explored. The study highlighted that in patients with MKD, the incidence of adverse drug reactions (ADR) was 42.86%, with pyrexia being the most common ADR. Other ADRs included oropharyngeal pain and inflammation of the upper respiratory tract. Importantly, no serious adverse reactions, deaths, or new safety concerns were identified in patients with MKD during the study period. Furthermore, a case of isolated hyperbilirubinemia was reported in a patient with MKD after initial treatment, which was not serious and was reported as ‘recovered.’ This study also highlights the potential of canakinumab as a treatment option for MKD, while also highlighting the need to carefully monitor potential ADRs (68).

Currently, there is no consensus on the most effective canakinumab dosage regimen for patients with MKD. Certain patients appear to require higher doses or more frequent dose intervals to achieve and maintain a complete response. Additional research is necessary to determine the optimal dose for MKD patients and identify factors for personalised treatment.

There has been no direct comparison between anakinra and CAN. Researchers conducted a retrospective study on 13 adult patients with MKD who had received interleukin 1 (IL-1) blockade treatment. The study found that anakinra resulted in complete or partial remission in some patients, while CAN resulted in a better overall response. The study suggests that the patient’s genotype can influence therapeutic results, and more research is needed to personalise treatment for patients with MKD (66).

Without strong evidence, some experts use a combination therapy of canakinumab and on-demand anakinra for MKD patients who show an insufficient response to canakinumab alone.

For patients who do not respond adequately to interleukin-1 inhibitors, tumour necrosis factor (TNF) blockers have emerged as potential second-line agents. Their role in modulating inflammation suggests that they may be beneficial in the management of symptoms of MKD, although more extensive clinical data are needed (22, 64, 74).

Tocilizumab, an IL-6 receptor antagonist, has potential as a therapeutic option for MKD. However, evidence is limited to case reports. Caution should be exercised in its application until further research validates its efficacy and safety for this patient population (75, 76).

Statins, especially simvastatin, have been identified for their potential to curtail MKD attacks (77). Statins inhibit HMG-CoA reductase, an enzyme that functions upstream of mevalonate kinase in the mevalonate pathway. By inhibiting this enzyme, statins reduce mevalonate production. In the context of MKD, where there is accumulation of mevalonate due to the deficiency of mevalonate kinase, statins could reduce this accumulation, thus decreasing the toxicity associated with elevated mevalonate levels.

However, the reduction in statin mevalonate production also decreases downstream isoprenoids. Since isoprenoid deficiency is believed to be a significant factor in the inflammatory symptoms seen in MKD, the use of statins could exacerbate this deficiency. Interestingly, in a single patient exhibiting the MA phenotype, administration of a statin appeared to trigger a severe attack (78). The SHARE guideline does not recommend the use of statins in the treatment of MKD, likely due to the potential complications arising from further isoprenoid depletion (31). Interestingly, neither the EULAR/ACR nor the German guidelines discuss the role of statins in the treatment of MKD, suggesting a consensus on the limited therapeutic potential of statins in this context (47, 48).

Colchicine is occasionally used by some experts in mild cases of MKD and in patients with VUS, based on limited case reports, including one involving an 8-month-old infant with an atypical MKD presentation who responded well to colchicine treatment over 8 months without further fever episodes (79).

A case report highlighted the use of bisphosphonates, specifically alendronate, in a patient with MKD. Bisphosphonates, which typically prevent bone resorption by inhibiting farnesyl diphosphate synthase, act downstream in the mevalonate pathway compared to mevalonate kinase. Theoretically, alendronate should exacerbate biochemical abnormalities in MKD, making it seem contraindicated. However, treatment was initiated to counteract a severe reduction in bone mineral density (BMD) due to glucocorticoids, despite *in vitro* studies suggesting that bisphosphonates could intensify fever attacks in MKD by further disrupting an already compromised metabolic pathway. Contrary to expectations, the patient experienced complete remission of symptoms of MKD and laboratory abnormalities after alendronate treatment (80). Despite the promising results reported by Cantarini et al. in their case study, recent research could not replicate the anti-inflammatory benefits of alendronate *in vitro* using both a murine cell line and monocytes extracted from two patients with MKD (81).

Beneficial inhibition of Janus kinase by tofacitinib has been reported in a few patients with familial mediterranean fever patients (82–84). However, there are no substantial data on JAK inhibition in MKD to inform guidance.

For patients with severe and refractory MKD, haematopoietic stem cell transplantation (HSCT) has been considered a ‘last resort’ option. It offers the potential for a definitive cure by replacing the defective immune system. However, HSCT is associated with significant risks and is reserved for the most severe cases where other treatments have failed (85–89). In a multicentre retrospective analysis by Jeyaratnam et al., allogeneic haematopoietic stem cell transplantation (HSCT) results were evaluated in patients with MKD (90). Of the nine patients analysed, seven achieved complete remission and were symptom-free during follow-up. However, two patients experienced transplant-related complications that resulted in death. The study underscores that while HSCT may be effective in patients with severely affected MKD, there is notable treatment-related morbidity and mortality. The authors advocate for the consideration of transplantation in patients who do not respond to conservative therapy, given the course of the severe disease.

Supportive measures also play a crucial role in the overall management of MKD. The guidelines focus on addressing potential triggers, patient education, monitoring, and lifestyle modifications. The 2013 SHARE guideline, in particular, underscores the importance of patient education and addressing potential triggers (31).

### Treat-to-target strategies

The primary objective of MKD treatment, as highlighted in both the German and EULAR/ACR guidelines, is to achieve sustained remission or, at a minimum, a significant decrease in the frequency and severity of inflammatory episodes (47, 48). This approach provides immediate symptomatic relief and prevents long-term complications, thus improving the patient’s overall quality of life (**Table 3**). The German PRO-KIND consensus statements and the resulting clinical treatment pathways (CTP) represent a significant evolution that transcends the disease classification-centric recommendations of the 2013 SHARE guideline (**Table 4**). This advancement underscores the dynamic understanding and approach to effectively managing MKD (47).

Routine evaluations, including clinical examinations and laboratory diagnostics, are crucial. These evaluations, together with patient and physician reports, provide a holistic view of therapeutic efficacy, facilitating timely treatment adjustments. To gauge disease activity, tools such as daily symptoms diary scores, the Autoinflammatory Disease Activity Index (AIDAI), the Physician’s Global Assessment (PGA), and the Patient-Parent Global Assessment (PPGA) should be followed (91). In a study by Koné-Paut et al., the feasibility of AIDAI was specifically evaluated for patients with MKD who underwent CAN treatment. Patients were required to complete the AIDAI questionnaire daily. The findings highlighted that AIDAI effectively distinguished between active and inactive states of MKD, underscoring its potential as a valuable tool in clinical settings to monitor disease activity and therapeutic outcomes in patients with MKD (92).

Similarly, ter Haar et al. developed the Autoinflammatory Disease Damage Index (ADDI) to assess persistent or irreversible changes in structure or function present for at least 6 months not attributable to ongoing disease activity in FMF, CAPS, TRAPS, and MKD (93). Based on a consensus, 18 preliminary items were chosen in eight organ systems and scored. ADDI was later validated using 110 clinical cases on paper by 37 experts and was found to be a reliable and valid instrument to quantify damage, thus contributing to the measurement of standardised disease outcome measurement (94).

If the ideal state of remission remains unattainable, achieving minimal disease activity is an alternative therapeutic target. The precise definitions of remission and minimal disease activity, along with their validation, are currently key research areas in the field of autoinflammatory diseases. In the absence of a definitive mathematical formula to seamlessly integrate various outcome parameters, consistent estimates of disease activity undeniably support decisions to modify therapies. However, inconsistent estimates have the same significance, necessitating further in-depth evaluations.

If therapeutic goals are not achieved within a specified time frame or adverse events emerge, it becomes essential to re-evaluate and potentially alter the treatment approach. Both guidelines advocate a stepwise strategy in which the intensity of the treatment increases if the initial interventions prove to be insufficient.

Biologic agents, especially IL-1 inhibitors such as anakinra and canakinumab, are recommended for the introduction or escalation of therapy when conventional treatments are inadequate. A combination of clinical insights, patient preferences, and disease severity influences the decision to increase treatment.

On the contrary, when a patient exhibits prolonged remission, there may be a window to decrease the intensity of treatment. However, such de-escalation should be cautiously approached, ensuring vigilant monitoring to swiftly detect and manage potential flare-ups.

A comprehensive treatment perspective is a central theme in both guidelines. This includes meticulous consideration of associated conditions and possible disease triggers and a pronounced emphasis on patient education and active participation in treatment decisions.

## Vaccination

Vaccination in patients with MKD is a delicate balance between mitigating flare risks and preventing infectious diseases. Despite the lack of clear evidence, it is crucial to maintain vaccination schedules, with adjustments guided by immunomodulatory treatments and expert consultation. An important question is whether patients on maintenance biological therapy can continue treatment while receiving live vaccines and how to manage vaccine-induced shubs. In a case report in which anakinra was used 72 hours after a vaccine-induced febrile episode, adequate seroconversion was observed (95). A recent paper focused on evaluating the immune response after COVID-19 vaccination in patients with systemic autoinflammatory diseases receiving IL-1 inhibitors (IL-1i) and comparing it with the response in healthy, vaccinated individuals. The results indicated that the immune response in patients treated with IL-1i was similar to that of healthy controls. Furthermore, no adverse effects were reported after vaccination, and IL-1i administration was maintained before and after the vaccine to prevent any exacerbation of symptoms (96). In the context of patients with MKD who are being vaccinated, the expert panel recommends that IL-1 inhibitors (IL-1i) should be continued throughout the vaccination process. We believe that the risk of live vaccine-mediated disease in patients on IL-1 blockade is largely theoretical. Anakinra, a specific IL-1 receptor antagonist, has been shown to not alter vaccination responses and can be administered safely around the time of vaccination. Furthermore, we suggest that using anakinra on demand to facilitate vaccination is an effective strategy to manage and prevent potential flare-ups, ensuring that patients receive the full benefit of immunisation without disrupting their ongoing treatment.

## Data Availability

The original contributions presented in the study are included in the article/**Supplementary Material**. Further inquiries can be directed to the corresponding author.

## References

[1] van der Burgh R ter Haar NM Boes ML Frenkel J . Mevalonate kinase deficiency, a metabolic autoinflammatory disease. Clin Immunol. (2013) 147:197–206. doi: 10.1016/j.clim.2012.09.011 23110805

[2] Lainka E Neudorf U Lohse P Timmann C Bielak M Stojanov S . Incidence and clinical features of hyperimmunoglobulinemia D and periodic fever syndrome (HIDS) and spectrum of mevalonate kinase (MVK) mutations in German children. Rheumatol Int. (2012) 32:3253–60. doi: 10.1007/s00296-011-2180-8 22038276

[3] Project EE autoinflammatory collaborating group for the PRITO (PRINTO) and E Toplak N Dolezalovà P Constantin T Sedivà A Pašić S . Periodic fever syndromes in Eastern and Central European countries: results of a pediatric multinational survey. Pediatr Rheumatol. (2010) 8:29. doi: 10.1186/1546-0096-8-29 PMC3014922 21539753

[4] Zhang S . Natural history of mevalonate kinase deficiency: a literature review. Pediatr Rheumatol Online J. (2016) 14:30. doi: 10.1186/s12969-016-0091-7 27142780 PMC4855321

[5] Govindaraj GM Jain A Peethambaran G Bhoyar RC Vellarikkal SK Ganapati A . Spectrum of clinical features and genetic variants in mevalonate kinase (MVK) gene of South Indian families suffering from Hyperimmunoglobulin D Syndrome. PloS One. (2020) 15:e0237999. doi: 10.1371/journal.pone.0237999 32822427 PMC7442240

[6] Tanaka T Yoshioka K Nishikomori R Sakai H Abe J Yamashita Y . National survey of Japanese patients with mevalonate kinase deficiency reveals distinctive genetic and clinical characteristics. Mod Rheumatol. (2019) 29:181–7. doi: 10.1080/14397595.2018.1442639 29451047

[7] Houten SM Kuis W Duran M de Koning TJ van Royen-Kerkhof A Romeijn GJ . Mutations in MVK, encoding mevalonate kinase, cause hyperimmunoglobulinaemia D and periodic fever syndrome. Nat Genet. (1999) 22:175–7. doi: 10.1038/9691 10369261

[8] Drenth JPH Cuisset L Grateau G Vasseur C van de Velde-Visser SD de Jong JGN . Mutations in the gene encoding mevalonate kinase cause hyper-IgD and periodic fever syndrome. Nat Genet. (1999) 22:178–81. doi: 10.1038/9696 10369262

[9] Frenkel J Simon A . (2019) Mevalonate Kinase Deficiency in Hashkes PJ et al. (eds), Textbook of Autoinflammation. Springer: Springer Nature Switzerland AG. 315-328. doi: 10.1007/978-3-319-98605-0_18.

[10] Papa R Doglio M Lachmann HJ Ozen S Frenkel J Simon A . A web-based collection of genotype-phenotype associations in hereditary recurrent fevers from the Eurofever registry. Orphanet J Rare Dis. (2017) 12:167. doi: 10.1186/s13023-017-0720-3 29047407 PMC5648458

[11] Houten SM van Woerden CS Wijburg FA Wanders RJA Waterham HR . Carrier frequency of the V377I (1129G>A) MVK mutation, associated with Hyper-IgD and periodic fever syndrome, in the Netherlands. Eur J Hum Genet. (2003) 11:196–200. doi: 10.1038/sj.ejhg.5200933 12634869

[12] Browne C Timson DJ . In silico prediction of the effects of mutations in the human mevalonate kinase gene: towards a predictive framework for mevalonate kinase deficiency. Ann Hum Genet. (2015) 79:451–9. doi: 10.1111/ahg.12126 26420133

[13] Houten SM Frenkel J Rijkers GT Wanders RJA Kuis W Waterham HR . Temperature dependence of mutant mevalonate kinase activity as a pathogenic factor in Hyper-IgD and periodic fever syndrome. Hum Mol Genet. (2002) 11:3115–24. doi: 10.1093/hmg/11.25.3115 12444096

[14] Tricarico PM Kleiner G Piscianz E Zanin V Monasta L Crovella S . Temperature and drug treatments in mevalonate kinase deficiency: an ex vivo study. BioMed Res Int. (2013) 2013:715465. doi: 10.1155/2013/715465 24073415 PMC3773414

[15] Mezzavilla M Moura RR Celsi F Tricarico PM Crovella S . MMAB, a novel candidate gene to be screened in the molecular diagnosis of Mevalonate Kinase Deficiency. Rheumatol Int. (2018) 38:121–7. doi: 10.1007/s00296-017-3890-3 29234874

[16] Jeyaratnam J ter Haar NM de Sain-van der Velden MGM Waterham HR van Gijn ME Frenkel J . Diagnostic value of urinary mevalonic acid excretion in patients with a clinical suspicion of mevalonate kinase deficiency. JIMD Rep. (2016) 27:33–8. doi: 10.1007/8904_2015_489 PMC4867845 26409462

[17] Stabile A Compagnone A Napodano S Raffaele CGL Patti M Rigante D . Mevalonate kinase genotype in children with recurrent fevers and high serum IgD level. Rheumatol Int. (2013) 33:3039–42. doi: 10.1007/s00296-012-2577-z 23239036

[18] Brennenstuhl H Nashawi M Schröter J Baronio F Beedgen L Gleich F . Phenotypic diversity, disease progression, and pathogenicity of MVK missense variants in mevalonic aciduria. J Inherit Metab Dis. (2021) 44:1272–87. doi: 10.1002/jimd.12412 34145613

[19] Rodrigues F Philit J-B Giurgea I Anglicheau D Roux J-J Hoyeau N . AA amyloidosis revealing mevalonate kinase deficiency: A report of 20 cases including two new French cases and a comprehensive review of literature. Semin Arthritis Rheum. (2020) 50:1370–3. doi: 10.1016/j.semarthrit.2020.03.005 32252977

[20] Atzmony L Choate KA . Second-hit somatic mutations in mevalonate pathway genes underlie porokeratosis. J Investig Dermatol. (2019) 139:2409–11. doi: 10.1016/j.jid.2019.07.723 PMC7962864 31753123

[21] Kellner U Stöhr H Weinitz S Farmand G Weber BHF . Mevalonate kinase deficiency associated with ataxia and retinitis pigmentosa in two brothers with MVK gene mutations. Ophthalmic Genet. (2017) 38:340–4. doi: 10.1080/13816810.2016.1227459 28095071

[22] ter Haar NM Jeyaratnam J Lachmann HJ Simon A Brogan PA Doglio M . The phenotype and genotype of mevalonate kinase deficiency: A series of 114 cases from the eurofever registry. Arthritis Rheumatol. (2016) 68:2795–805. doi: 10.1002/art.39763 27213830

[23] Kubo A Sasaki T Suzuki H Shiohama A Aoki S Sato S . Clonal expansion of second-hit cells with somatic recombinations or C>T transitions form porokeratosis in MVD or MVK mutant heterozygotes. J Invest Dermatol. (2019) 139:2458–2466.e9. doi: 10.1016/j.jid.2019.05.020 31207227

[24] Zhang Z Li C Wu F Ma R Luan J Yang F . Genomic variations of the mevalonate pathway in porokeratosis. eLife. (2015) 4:e06322. doi: 10.7554/elife.06322 26202976 PMC4511816

[25] Boursier G Rittore C Milhavet F Cuisset L Touitou I . Mevalonate kinase-associated diseases: hunting for phenotype-genotype correlation. J Clin Med. (2021) 10:1552. doi: 10.3390/jcm10081552 33917151 PMC8067830

[26] Carapito R Carapito C Morlon A Paul N Jacome ASV Alsaleh G . Multi-OMICS analyses unveil STAT1 as a potential modifier gene in mevalonate kinase deficiency. Ann Rheum Dis. (2018) 77:1675–87. doi: 10.1136/annrheumdis-2018-213524 PMC6225799 30030262

[27] Moura R Tricarico PM Coelho AVC Crovella S . GRID2 a novel gene possibly associated with mevalonate kinase deficiency. Rheumatol Int. (2015) 35:657–9. doi: 10.1007/s00296-014-3115-y 25146332

[28] Bader-Meunier B Martins AL Charbit-Henrion F Meinzer U Belot A Cuisset L . Mevalonate kinase deficiency: A cause of severe very-early-onset inflammatory bowel disease. Inflammation Bowel Dis. (2021) 27:1853–7. doi: 10.1093/ibd/izab139 34525209

[29] Bianco AM Girardelli M Vozzi D Crovella S Kleiner G Marcuzzi A . Mevalonate kinase deficiency and IBD: shared genetic background. Gut. (2014) 63:1367–8. doi: 10.1136/gutjnl-2013-306555 PMC4112436 24531851

[30] Ben-Chetrit E Gattorno M Gul A Kastner DL Lachmann HJ Touitou I . Consensus proposal for taxonomy and definition of the autoinflammatory diseases (AIDs): a Delphi study. Ann Rheum Dis. (2018) 77:1558. doi: 10.1136/annrheumdis-2017-212515 30100561

[31] ter Haar NM Oswald M Jeyaratnam J Anton J Barron KS Brogan PA . Recommendations for the management of autoinflammatory diseases. Ann Rheum Dis. (2015) 74:1636–44. doi: 10.1136/annrheumdis-2015-207546 26109736

[32] Burns PB Rohrich RJ Chung KC . The levels of evidence and their role in evidence-based medicine. Plast Reconstr Surg. (2011) 128:305–10. doi: 10.1097/prs.0b013e318219c171 PMC3124652 21701348

[33] van der Hilst JCH Drenth JPH Bodar EJ Bijzet J van der Meer JWM Simon A . Serum amyloid A serum concentrations and genotype do not explain low incidence of amyloidosis in Hyper-IgD syndrome. Amyloid. (2005) 12:115–9. doi: 10.1080/13506120500106982 16011988

[34] Liang P Huang Q Xu Y Chen L Li J Xu A . High serum immunoglobulin D levels in systemic lupus erythematosus: more to be found? Clin Rheumatol. (2023) 42:1069–76. doi: 10.1007/s10067-022-06457-9 36585530

[35] Simon A Cuisset L Vincent M-F van der Velde-Visser SD Delpech M van der Meer JWM . Molecular analysis of the mevalonate kinase gene in a cohort of patients with the hyper-igD and periodic fever syndrome: its application as a diagnostic tool. Ann Intern Med. (2001) 135:338. doi: 10.7326/0003-4819-135-5-200109040-00010 11529697

[36] Ammouri W Cuisset L Rouaghe S Rolland M-O Delpech M Grateau G . Diagnostic value of serum immunoglobulinaemia D level in patients with a clinical suspicion of hyper IgD syndrome. Rheumatology. (2007) 46:1597–600. doi: 10.1093/rheumatology/kem200 17804452

[37] Simon A Bijzet J Voorbij HAM Mantovani A Meer JWMVD Drenth JPH . Effect of inflammatory attacks in the classical type hyper-IgD syndrome on immunoglobulin D, cholesterol and parameters of the acute phase response. J Intern Med. (2004) 256:247–53. doi: 10.1111/j.1365-2796.2004.01359.x 15324368

[38] Haraldsson Á Weemaes CMR de Boer AW Bakkeren JAJM Stoelinga GBA . Immunological studies in the hyper-immunoglobulin D syndrome. J Clin Immunol. (1992) 12:424–8. doi: 10.1007/bf00918854 1287034

[39] Klasen IS Göertz JHC van de Wiel GAS Weemaes CMR van der Meer JWM Drenth JPH . Hyper-immunoglobulin A in the hyperimmunoglobulinemia D syndrome. Clin Diagn Lab Immunol. (2001) 8:58–61. doi: 10.1128/cdli.8.1.58-61.2001 11139196 PMC96011

[40] Reitzle L Maier B Stojanov S Teupser D Muntau AC Vogeser M . Quantification of mevalonate-5-phosphate using UPLC-MS/MS for determination of mevalonate kinase activity. Clin Biochem. (2015) 48:781–7. doi: 10.1016/j.clinbiochem.2015.05.007 25982894

[41] Munoz MA Jurczyluk J Mehr S Chai RC Arts RJW Sheu A . Defective protein prenylation is a diagnostic biomarker of mevalonate kinase deficiency. J Allergy Clin Immunol. (2017) 140:873–875.e6. doi: 10.1016/j.jaci.2017.02.033 28501347

[42] Jurczyluk J Munoz MA Skinner OP Chai RC Ali N Palendira U . Mevalonate kinase deficiency leads to decreased prenylation of Rab GTPases. Immunol Cell Biol. (2016) 94:994–9. doi: 10.1038/icb.2016.58 PMC5122740 27377765

[43] Munoz MA Jurczyluk J Simon A Hissaria P Arts RJW Coman D . Defective protein prenylation in a spectrum of patients with mevalonate kinase deficiency. Front Immunol. (2019) 10:1900. doi: 10.3389/fimmu.2019.01900 31474985 PMC6702261

[44] Gattorno M Sormani MP D’Osualdo A Pelagatti MA Caroli F Federici S . A diagnostic score for molecular analysis of hereditary autoinflammatory syndromes with periodic fever in children. Arthritis Rheumatism. (2008) 58:1823–32. doi: 10.1002/art.23474 18512793

[45] Wulffraat NM Vastert B consortium S . Time to share. Pediatr Rheumatol. (2013) 11:5. doi: 10.1186/1546-0096-11-5 PMC3583675 23414510

[46] Federici S Sormani MP Ozen S Lachmann HJ Amaryan G Woo P . Evidence-based provisional clinical classification criteria for autoinflammatory periodic fevers. Ann Rheum Dis. (2015) 74:799–805. doi: 10.1136/annrheumdis-2014-206580 25637003

[47] Hansmann S Lainka E Horneff G Holzinger D Rieber N Jansson AF . Consensus protocols for the diagnosis and management of the hereditary autoinflammatory syndromes CAPS, TRAPS and MKD/HIDS: a German PRO-KIND initiative. Pediatr Rheumatol Online J. (2020) 18:17. doi: 10.1186/s12969-020-0409-3 32066461 PMC7027082

[48] Romano M Arici ZS Piskin D Alehashemi S Aletaha D Barron KS . The 2021 EULAR/American College of Rheumatology points to consider for diagnosis, management and monitoring of the interleukin-1 mediated autoinflammatory diseases: cryopyrin-associated periodic syndromes, tumour necrosis factor receptor-associated periodic syndrome, mevalonate kinase deficiency, and deficiency of the interleukin-1 receptor antagonist. Ann Rheum Dis. (2022) 81:907–21. doi: 10.1136/annrheumdis-2021-221801 35623638

[49] McNaughton P Willcocks S Lum SH Whitehead B Peake J Preece K . Making a diagnosis of periodic fever syndrome: Experience from a single tertiary centre. J Paediatr Child Health. (2022) 58:404–8. doi: 10.1111/jpc.15722 34499401

[50] Gattorno M Hofer M Federici S Vanoni F Bovis F Aksentijevich I . Classification criteria for autoinflammatory recurrent fevers. Ann Rheum Dis. (2019) 78:1025–32. doi: 10.1136/annrheumdis-2019-215048 31018962

[51] Dingulu G Georgin-Lavialle S Koné-Paut I Pillet P Pagnier A Merlin E . Validation of the new classification criteria for hereditary recurrent fever in an independent cohort: experience from the JIR Cohort Database. Rheumatol (Oxford). (2020) 59:2947–52. doi: 10.1093/rheumatology/keaa031 32125423

[52] Çağlayan Ş Mardinoğlu G Yarar MH Ulu K Coşkuner T Yiğit RE . The assessment of autoinflammatory disease classification criteria (Eurofever/PRINTO) in a real-life cohort. Clin Rheumatol. (2023) 42:1645–53. doi: 10.1007/s10067-023-06557-0 36826737

[53] Infevers . Available online at: https://infevers.umai-montpellier.fr/web/ (Accessed August 16, 2023).

[54] Hoang TK Albert DA . Novel presentations of periodic fever syndromes: Discrepancies between genetic and clinical diagnoses. Eur J Rheumatol. (2019) 6:12–8. doi: 10.5152/eurjrheum.2018.18023 PMC6459325 30407166

[55] Shinar Y Obici L Aksentijevich I Bennetts B Austrup F Ceccherini I . Guidelines for the genetic diagnosis of hereditary recurrent fevers. Ann Rheum Dis. (2012) 71:1599. doi: 10.1136/annrheumdis-2011-201271 22661645 PMC3500529

[56] Shinar Y Ceccherini I Rowczenio D Aksentijevich I Arostegui J Ben-Chétrit E . ISSAID/EMQN best practice guidelines for the genetic diagnosis of monogenic autoinflammatory diseases in the next-generation sequencing era. Clin Chem. (2020) 66:525–36. doi: 10.1093/clinchem/hvaa024 32176780

[57] Lachmann HJ . Periodic fever syndromes. Best Pract Res Clin Rheumatol. (2017) 31:596–609. doi: 10.1016/j.berh.2017.12.001 29773275

[58] Sutera D Bustaffa M Papa R Matucci-Cerinic C Matarese S D’Orsi C . Clinical characterization, long-term follow-up, and response to treatment of patients with syndrome of undifferentiated recurrent fever (SURF). Semin Arthritis Rheum. (2022) 55:152024. doi: 10.1016/j.semarthrit.2022.152024 35598507

[59] Papa R Rusmini M Volpi S Caorsi R Picco P Grossi A . Next generation sequencing panel in undifferentiated autoinflammatory diseases identifies patients with colchicine-responder recurrent fevers. Rheumatology. (2020) 59:344–60. doi: 10.1093/rheumatology/kez270 31325311

[60] Alexeeva E Shingarova M Dvoryakovskaya T Lomakina O Fetisova A Isaeva K . Safety and efficacy of canakinumab treatment for undifferentiated autoinflammatory diseases: the data of a retrospective cohort two-centered study. Front Med. (2023) 10:1257045. doi: 10.3389/fmed.2023.1257045 PMC10685903 38034538

[61] Garg S Wynne K Omoyinmi E Eleftheriou D Brogan P . Efficacy and safety of anakinra for undifferentiated autoinflammatory diseases in children: a retrospective case review. Rheumatol Adv Pr. (2019) 3:rkz004. doi: 10.1093/rap/rkz004 PMC6649913 31431992

[62] Luu I Nation J Page N Carvalho D Magit A Jiang W . Undifferentiated recurrent fevers in pediatrics are clinically distinct from PFAPA syndrome but retain an IL-1 signature. Clin Immunol. (2021) 226:108697. doi: 10.1016/j.clim.2021.108697 33636366 PMC8089050

[63] Casa FD Vitale A Lopalco G Ruscitti P Ciccia F Emmi G . Development and implementation of the AIDA international registry for patients with undifferentiated systemic autoInflammatory diseases. Front Med. (2022) 9:908501. doi: 10.3389/fmed.2022.908501 PMC9226373 35755024

[64] Kostjukovits S Kalliokoski L Antila K Korppi M . Treatment of hyperimmunoglobulinemia D syndrome with biologics in children: review of the literature and Finnish experience. Eur J Pediatr. (2015) 174:707–14. doi: 10.1007/s00431-015-2505-9 25721923

[65] Bodar EJ Kuijk LM Drenth JPH van der Meer JWM Simon A Frenkel J . On-demand anakinra treatment is effective in mevalonate kinase deficiency. Ann Rheum Dis. (2011) 70:2155. doi: 10.1136/ard.2011.149922 21859689

[66] Deshayes S Georgin-Lavialle S Hot A Durel C-A Hachulla E Rouanes N . Efficacy of continuous interleukin 1 blockade in mevalonate kinase deficiency: A multicenter retrospective study in 13 adult patients and literature review. J Rheumatol. (2018) 45:425–9. doi: 10.3899/jrheum.170684 29335349

[67] Rossi-Semerano L Fautrel B Wendling D Hachulla E Galeotti C Semerano L . Tolerance and efficacy of off-label anti-interleukin-1 treatments in France: a nationwide survey. Orphanet J Rare Dis. (2015) 10:19. doi: 10.1186/s13023-015-0228-7 25758134 PMC4340831

[68] Hosono K Matsumoto K Shimbo M Tsumiyama I Kato C . Real-world safety and effectiveness of canakinumab in patients with tumour necrosis factor receptor-associated periodic syndrome or hyperimmunoglobulinaemia D syndrome: Interim results from post-marketing surveillance in Japan. Mod Rheumatol. (2023) 33:381–91. doi: 10.1093/mr/roac041 35575279

[69] Hur P Lomax KG Ionescu-Ittu R Manceur AM Xie J Cammarota J . Reasons for canakinumab initiation among patients with periodic fever syndromes: a retrospective medical chart review from the United States. Pediatr Rheumatol Online J. (2021) 19:143. doi: 10.1186/s12969-021-00605-2 34521444 PMC8439059

[70] Çakan M Karadağ ŞG Ayaz NA . Canakinumab in colchicine resistant familial Mediterranean fever and other pediatric rheumatic diseases. Turk J Pediatr. (2020) 62:167–74. doi: 10.24953/turkjped.2020.02.001 32419407

[71] Jeyaratnam J Simon A Calvo I Constantin T Shcherbina A Hofer M . Long-term efficacy and safety of canakinumab in patients with mevalonate kinase deficiency: results from the randomised Phase 3 CLUSTER trial. Rheumatol (Oxford). (2022) 61:2088–94. doi: 10.1093/rheumatology/keab696 34554243

[72] Lachmann HJ Lauwerys B Miettunen P Kallinich T Jansson A Rosner I . Canakinumab improves patient-reported outcomes in children and adults with autoinflammatory recurrent fever syndromes: results from the CLUSTER trial. Clin Exp Rheumatol. (2021) 39 Suppl 132:51–8. doi: 10.55563/clinexprheumatol/e92f7o 34622762

[73] Balci S Ekinci RMK Dogruel D Altintas DU Yilmaz M . Growth parameters of turkish children with an autoinflammatory disease before and after canakinumab treatment. Indian Pediatr. (2020) 57:637–40. doi: 10.1007/s13312-020-1892-9 32727940

[74] Ozen S Kuemmerle-Deschner JB Cimaz R Livneh A Quartier P Kone-Paut I . International retrospective chart review of treatment patterns in severe familial Mediterranean fever, tumor necrosis factor receptor-associated periodic syndrome, and mevalonate kinase deficiency/hyperimmunoglobulinemia D syndrome. Arthritis Care Res (Hoboken). (2017) 69:578–86. doi: 10.1002/acr.23120 27723279

[75] Shendi HM Devlin LA Edgar JD . Interleukin 6 blockade for hyperimmunoglobulin D and periodic fever syndrome. J Clin Rheumatol. (2014) 20:103–5. doi: 10.1097/01.rhu.0000442576.41537.de 24561416

[76] Musters A Tak PP Baeten DLP Tas SW . Anti-interleukin 6 receptor therapy for hyper-IgD syndrome. BMJ Case Rep. (2015) 2015):bcr2015210513. doi: 10.1136/bcr-2015-210513 PMC4636692 26516243

[77] Simon A Drewe E Meer JWM Powell RJ Kelley RI Stalenhoef AFH . Simvastatin treatment for inflammatory attacks of the hyperimmunoglobulinemia D and periodic fever syndrome. Clin Pharmacol Ther. (2004) 75:476–83. doi: 10.1016/j.clpt.2004.01.012 15116060

[78] Hoffmann GF Charpentier C Mayatepek E Mancini J Leichsenring M Gibson KM . Clinical and biochemical phenotype in 11 patients with mevalonic aciduria. Pediatrics. (1993) 91:915–21. doi: 10.1542/peds.91.5.915 8386351

[79] Yekedüz MK Doğulu N Öncül Ü Köse E Ceylaner S Eminoğlu FT . An atypical presentation of mevalonate kinase deficiency in response to colchicine treatment. Mol Syndromol. (2022) 13:146–51. doi: 10.1159/000518825 PMC8928187 35418827

[80] Cantarini L Vitale A Magnotti F Lucherini OM Caso F Frediani B . Weekly oral alendronate in mevalonate kinase deficiency. Orphanet J Rare Dis. (2013) 8:196. doi: 10.1186/1750-1172-8-196 24360083 PMC3880037

[81] Tricarico PM Girardelli M Kleiner G Knowles A Valencic E Crovella S . Alendronate, a double-edged sword acting in the mevalonate pathway. Mol Med Rep. (2015) 12:4238–42. doi: 10.3892/mmr.2015.3957 PMC4526081 26096667

[82] Gök K Cengiz G Erol K Ozgocmen S . Tofacitinib suppresses disease activity and febrile attacks in a patient with coexisting rheumatoid arthritis and familial Mediterranean fever. Acta Reum Port. (2017) 42:88–90. 28371574

[83] Karadeniz H Güler AA Atas N Satış H Salman RB Babaoglu H . Tofacitinib for the treatment for colchicine-resistant familial Mediterranean fever: case-based review. Rheumatol Int. (2020) 40:169–73. doi: 10.1007/s00296-019-04490-7 31813060

[84] Garcia-Robledo JE Aragón CC Nieto-Aristizabal I Posso-Osorio I Cañas CA Tobón GJ . Tofacitinib for familial Mediterranean fever: a new alternative therapy? Rheumatology. (2019) 58:553–4. doi: 10.1093/rheumatology/key384 30535114

[85] Neven B Valayannopoulos V Quartier P Blanche S Prieur A-M Debré M . Allogeneic bone marrow transplantation in mevalonic aciduria. N Engl J Med. (2007) 356:2700–3. doi: 10.1056/nejmoa070715 17596604

[86] Lambros AM Sagar KA Dahlgren MK Kosereisoglu D El-Abboud C Smith RT . CannaCount: an improved metric for quantifying estimates of maximum possible cannabinoid exposure. Sci Rep. (2023) 13:5869. doi: 10.1038/s41598-023-32671-9 37041309 PMC10090150

[87] Erdol S Cekic S Kılıc SC Saglam H Kılıc SS . Massive ascites in a canakinumab resistant case with MVA leading to bone marrow transplantation. Rheumatol Int. (2016) 36:1011–3. doi: 10.1007/s00296-016-3456-9 26965418

[88] Arkwright PD Abinun M Cant AJ . Mevalonic aciduria cured by bone marrow transplantation. N Engl J Med. (2007) 357:1350–0. doi: 10.1056/nejmc072018 17898110

[89] Faraci M Giardino S Podestà M Pierri F Dell’Orso G Beccaria A . Haploidentical α/β T-cell and B-cell depleted stem cell transplantation in severe mevalonate kinase deficiency. Rheumatol (Oxford). (2021) 60:4850–4. doi: 10.1093/rheumatology/keaa912 33410495

[90] Jeyaratnam J Faraci M Gennery AR Drabko K Algeri M Morimoto A . The efficacy and safety of allogeneic stem cell transplantation in Mevalonate Kinase Deficiency. Pediatr Rheumatol Online J. (2022) 20:56. doi: 10.1186/s12969-022-00716-4 35906690 PMC9338460

[91] Piram M Koné-Paut I Lachmann HJ Frenkel J Ozen S Kuemmerle-Deschner J . Validation of the auto-inflammatory diseases activity index (AIDAI) for hereditary recurrent fever syndromes. Ann Rheum Dis. (2014) 73:2168–73. doi: 10.1136/annrheumdis-2013-203666 PMC4251184 24026675

[92] Koné-Paut I Piram M Benseler S Kuemmerle-Deschner JB Jansson A Rosner I . Use of the Auto-inflammatory Disease Activity Index to monitor disease activity in patients with colchicine-resistant Familial Mediterranean Fever, Mevalonate Kinase Deficiency, and TRAPS treated with canakinumab. Joint Bone Spine. (2022) 89:105448. doi: 10.1016/j.jbspin.2022.105448 35944600

[93] ter Haar NM Annink KV Al-Mayouf SM Amaryan G Anton J Barron KS . Development of the autoinflammatory disease damage index (ADDI). Ann Rheum Dis. (2017) 76:821–30. doi: 10.1136/annrheumdis-2016-210092 27811147

[94] ter Haar NM van Delft ALJ Annink KV van Stel H Al-Mayouf SM Amaryan G . In silico validation of the Autoinflammatory Disease Damage Index. Ann Rheum Dis. (2018) 77:1599–605. doi: 10.1136/annrheumdis-2018-213725 PMC8411437 30077992

[95] Bodar EJ van der Hilst JCH Drenth JPH van der Meer JWM Simon A . Effect of etanercept and anakinra on inflammatory attacks in the hyper-IgD syndrome: introducing a vaccination provocation model. Neth J Med. (2005) 63:260–4. 16093577

[96] Bindoli S Baggio C Galozzi P Vesentini F Doria A Cosma C . Autoinflammatory diseases and COVID-19 vaccination: analysis of SARS-coV-2 anti-S-RBD igG levels in a cohort of patients receiving IL-1 inhibitors. J Clin Med. (2023) 12:4741. doi: 10.3390/jcm12144741 37510856 PMC10380649

[97] Elhani I Hentgen V Grateau G Georgin-Lavialle S . Neurological manifestations in mevalonate kinase deficiency: A systematic review. Mol Genet Metab. (2022) 136:85–93. doi: 10.1016/j.ymgme.2022.04.006 35525811

[98] van der Hilst JCH Bodar EJ Barron KS Frenkel J Drenth JPH van der Meer JWM . Long-term follow-up, clinical features, and quality of life in a series of 103 patients with hyperimmunoglobulinemia D syndrome. Medicine. (2008) 87:301–10. doi: 10.1097/md.0b013e318190cfb7 19011501

[99] Yıldız Ç Yıldırım DG Inci A Tümer L Ergin FBC Yayla ENSS . A possibly new autoinflammatory disease due to compound heterozygous phosphomevalonate kinase gene mutation. Joint Bone Spine. (2023) 90:105490. doi: 10.1016/j.jbspin.2022.105490 36410683

[100] Berner J van de Wetering C Heredia RJ Rashkova C Ferdinandusse S Koster J . Phosphomevalonate kinase deficiency expands the genetic spectrum of systemic autoinflammatory diseases. J Allergy Clin Immunol. (2023) 152:1025–31. doi: 10.1016/j.jaci.2023.06.013 PMC10549927 37364720

[101] Jairaman A Badiger VA Raj S Nair KV Balan S Narayanan DL . A novel homozygous variant in PMVK is associated with enhanced IL1β secretion and a hyper-IgD syndrome-like phenotype. Clin Genet. (2024) 105:302–7. doi: 10.1111/cge.14451 38018277

[102] Wang J Liu Y Liu F Huang C Han S Lv Y . Loss-of-function mutation in PMVK causes autosomal dominant disseminated superficial porokeratosis. Sci Rep. (2016) 6:24226. doi: 10.1038/srep24226 27052676 PMC4823745

[103] Yıldırım DG Yıldırım ÇY Karaçayır N Şenol PE Yayla ENS Bakkaloğlu SA . Recurrent macrophage activation syndrome due to hyperimmunoglobulin D syndrome: a case-based review. Clin Rheumatol. (2023) 42:277–83. doi: 10.1007/s10067-022-06384-9 36149537

[104] Rigante D Emmi G Fastiggi M Silvestri E Cantarini L . Macrophage activation syndrome in the course of monogenic autoinflammatory disorders. Clin Rheumatol. (2015) 34:1333–9. doi: 10.1007/s10067-015-2923-0 25846831

[105] Rigante D Capoluongo E Bertoni B Ansuini V Chiaretti A Piastra M . First report of macrophage activation syndrome in hyperimmunoglobulinemia D with periodic fever syndrome. Arthritis Rheum. (2007) 56:658–61. doi: 10.1002/art.22409 17265501

[106] Bader-Meunier B Florkin B Sibilia J Acquaviva C Hachulla E Grateau G . Mevalonate kinase deficiency: A survey of 50 patients. Pediatrics. (2011) 128:e152–9. doi: 10.1542/peds.2010-3639 21708801

[107] Akula MK Shi M Jiang Z Foster CE Miao D Li AS . Control of the innate immune response by the mevalonate pathway. Nat Immunol. (2016) 17:922–9. doi: 10.1038/ni.3487 PMC4955724 27270400

