# Supplementary figures and images for: Mevalonate kinase deficiency: an updated clinical overview and revision of the SHARE recommendations
Source: Front Immunol. 2024 Nov 12;15:1466844. doi: 10.3389/fimmu.2024.1466844 (PMC11590122; doi:10.3389/fimmu.2024.1466844)

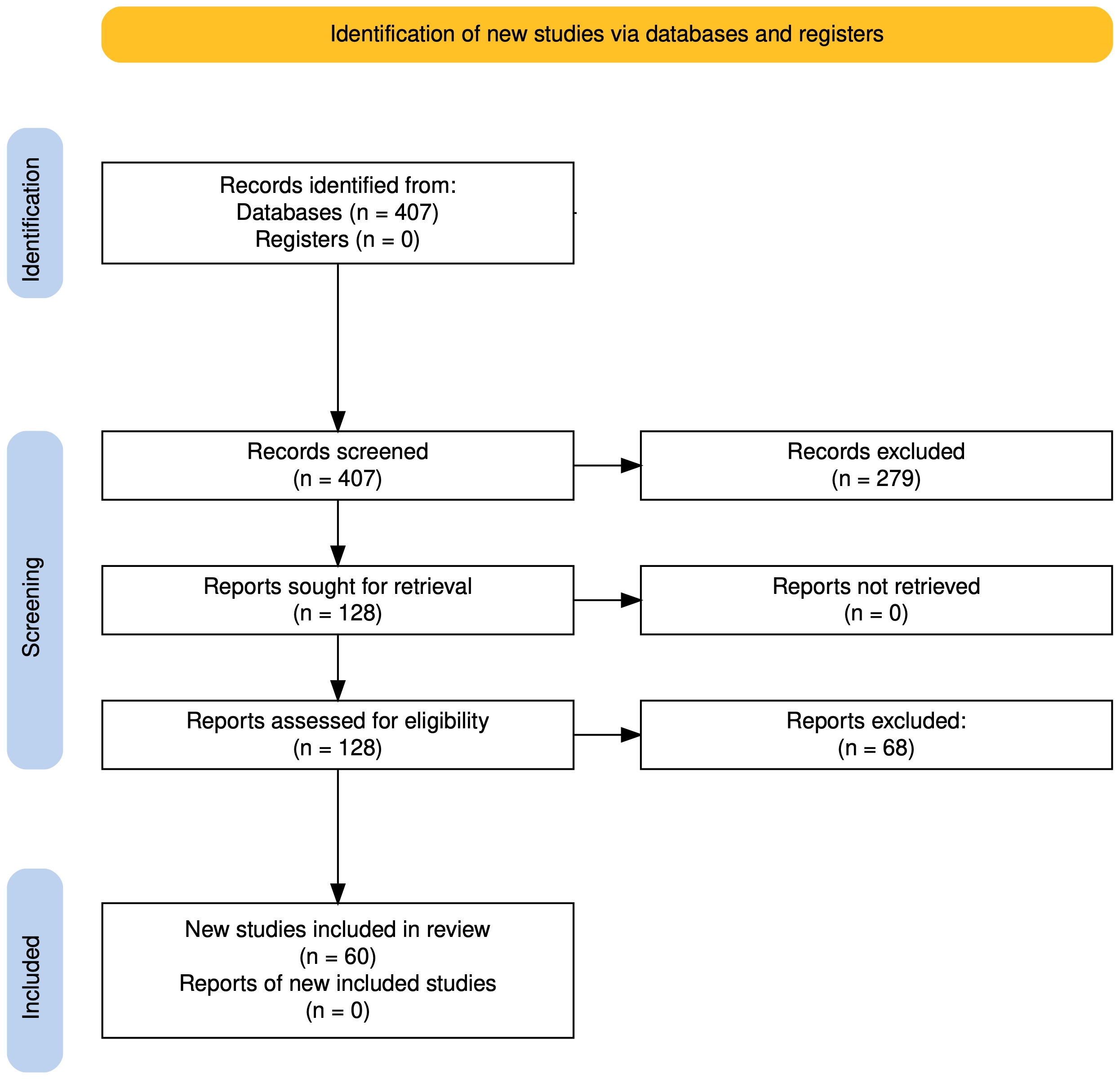

Supplement: Supplementary file 1 [file Image1.jpeg]
