# Supplementary material for: Mevalonate kinase deficiency: an updated clinical overview and revision of the SHARE recommendations
Source: Front Immunol. 2024 Nov 12;15:1466844. doi: 10.3389/fimmu.2024.1466844 (PMC11590122; doi:10.3389/fimmu.2024.1466844)
Supplement: Supplementary file 2 [file DataSheet1.pdf]

Supplementary Material

Search Strategy

A comprehensive search was conducted on PubMed, applying the following index terms and PICO framework to identify relevant articles related to MKD published after the last SHARE review. The search strategy was designed to ensure consistency with the previous review while capturing all new evidence available.

Index Terms: *"mevalonate kinase deficiency"[MeSH] OR MKD[tiab] OR "mevalonate kinase deficiency""[tiab]OR "mevalonicaciduria"[tiab] OR HIDS[tiab] OR HyperIgD[tiab] OR "hyperimmunoglobulin D"[tiab] OR "hyperimmunoglobulinemia D"[tiab] OR "hyperimmunoglobulinaemia D"[tiab] OR (Hyper[tiab] AND (IgD[tiab] OR "Ig D"[tiab] OR "immunoglobulin D"[tiab]))*

PICO:

P (Population):

- Population: Patients (mainly pediatric) with Mevalonate Kinase Deficiency (MKD)

I (Intervention):

- Interventions:
  - Diagnostic: Genetic testing (MVK gene), urinary mevalonic acid testing, inflammatory markers (CRP, SAA).
  - Therapeutic: Anti-inflammatory drugs (NSAIDs, corticosteroids), biologic agents (canakinumab, anakinra), supportive measures.
  - Guidelines: Implementation of the updated SHARE recommendations for the diagnosis and treatment of MKD.

C (Comparison):

- Comparisons:
  - Previous Guidelines: 2015 SHARE guidelines versus updated recommendations.
  - Standard Care: Conventional anti-inflammatory treatments (e.g., NSAIDs and corticosteroids) compared to biologic therapies like IL-1 inhibitors (canakinumab, anakinra).
  - Diagnostic Tools: Traditional clinical diagnosis versus advanced genetic and biochemical diagnostics.

O (Outcome):

- Outcomes:
  - Improved diagnostic accuracy for MKD.
  - Reduction in the frequency and severity of inflammatory episodes.
  - Better long-term patient outcomes, including growth, quality of life, and disease remission.
  - More personalized treatment plans based on genotype and clinical presentation.

Quality Assessment

We performed a comprehensive risk of bias analysis using the ROBINS-I tool. This assessment evaluated bias across several domains, including confounding, selection of participants, classification of interventions, deviations from intended interventions, missing data, measurement of outcomes, and selection of reported results. The detailed quality assessment can be accessed via [https://docs.google.com/spreadsheets/d/1uXThxDf4zGk9MMI2X24N0gG5UbsZu9X_n0TZnTDb6IM/edit?usp=sharing].

Validity Assessment

As described in the methods section of the manuscript, we adopted the Levels of Evidence and grading recommendations based on the hierarchical system outlined by Burns et al. Each study was classified according to Levels of Evidence, ranging from Level I (high-quality randomized controlled trials) to Level IV (expert opinion and case reports). The strength of recommendations was graded accordingly, with strong recommendations (Grade A) supported by Level I evidence or consistent findings from multiple studies at Levels II, III, or IV, and weaker recommendations (Grade C) based on inconsistent or lower-level evidence. The validity assessment was conducted by KT, LL, and MSZ. The full results of this validity assessment can be accessed via [https://docs.google.com/spreadsheets/d/1P8N2aZunPPB2ygzaBqab9uCyj4nQlPyTmBm48AF7Nig/edit?usp=sharing].

PRISMA flow diagram

The PRISMA flow chart is attached for your reference.
